# Genomic insight into the antibiotic-resistant determinants of *Escherichia coli* from small ruminants and manure in North West Province, South Africa

**DOI:** 10.3389/fmicb.2026.1950193

**Published:** 2026-09-28

**Authors:** Jody Howard, Oriel Thekisoe, Tsepo Ramatla, Kgaugelo E. Lekota

**Affiliations:** 1 Unit for Environmental Sciences and Management, North-West University, Potchefstroom, South Africa; 2 Department of Life Sciences, Center for Applied Food Safety and Biotechnology, Central University of Technology, Bloemfontein, South Africa

**Keywords:** antibiotic resistance, *Escherichia coli*, South Africa, virulence genes, whole genome sequencing

## Abstract

**Introduction:**

Livestock-associated *Escherichia coli* are important reservoirs of antimicrobial resistance and virulence determinants, yet comprehensive genomic data describing their genomic diversity and antibiotic resistance in South African small-ruminant production systems remain limited.

**Objective:**

Whole-genome sequencing was performed on 19 *E. coli* isolates recovered from sheep, goats, and manure collected from two farming systems in the North West Province, South Africa.

**Materials and methods:**

Genomic analyses revealed a dual pattern of clonal expansion and deep heterogeneity, with globally distributed sequence types (ST10, ST155) occurring alongside several novel or rarely reported STs. Comparative genomic analyses incorporating average nucleotide identity (ANI), multilocus sequence typing (MLST), *in silico* serotyping, antimicrobial resistance profiling, virulence characterization, and mobile genetic element analysis revealed substantial genomic diversity, comprising globally disseminated sequence types, including ST10 and ST155, together with several novel and infrequently reported lineages.

**Results:**

Although most isolates (*n* =17) belonged to phylogroups A and B1, the resistome was dominated by the multidrug efflux gene *mdf(A)* (95%) and *bla*_TEM − 1B_ (58%), corresponding with high phenotypic resistance to erythromycin (74%) and ampicillin (95%), while 26% of isolates exhibited multidrug-resistant phenotypes. Genomic analysis identified 71 plasmids representing diverse incompatibility groups and mobility classes, including conjugative and mobilizable IncHI1B and IncR plasmids carrying clinically relevant resistance genes. Approximately 89% belonged to commensal lineages (A and B1), while groups D and E were each represented by a single isolate.

**Conclusion:**

To our knowledge, this represents the first whole-genome sequencing-based genomic characterization of *E. coli* recovered from sheep and goats in South Africa, thereby expanding the known genomic diversity of livestock-associated *E. coli* within the country. Furthermore, data generated in this study emphasizes the necessity of ongoing genomic monitoring to track the emergence and spread of resistant *E. coli*, which is crucial for monitoring and mitigating potential public health risks.

## 1 Introduction

*Escherichia coli*, a member of the Enterobacteriaceae family and closely related to pathogens such as *Klebsiella, Salmonella* and *Serratia* species, serves as a model of a versatile bacterial species (Leimbach et al., 2013; Blount, 2015). It can cause both intestinal and extraintestinal diseases in humans and a wide range of animal hosts (Leimbach et al., 2013). Poultry, pigs, cattle, sheep, eggs, and milk are examples of livestock and animal-derived food products that have become important reservoirs for resistant strains of *E. coli* (Faridah et al., 2020; Ramatla et al., 2024a). The *E. coli* bacterium can survive in a variety of ecological settings, including soil, water, sediment, and food, but it usually exists as a benign commensal in the mucous layer of the colon and cecum (Leimbach et al., 2013). However, it can also cause fatal infections (Kaper et al., 2004; Sousa, 2006).

Pathogenic *E. coli* can cause urinary tract infections, sepsis/meningitis, and gastrointestinal diseases by either spreading throughout the body or remaining confined to mucosal surfaces (Nataro and Kaper, 1998). Pathogenic *E. coli* is broadly classified into intestinal pathogenic *E. coli* (IPEC) and extraintestinal pathogenic *E. coli* (ExPEC) (Leimbach et al., 2013). These include Shiga toxin–producing *E. coli* (STEC), enteropathogenic *E. coli* (EPEC), enterotoxigenic *E. coli* (ETEC), enteroinvasive *E. coli* (EIEC), enteroaggregative *E. coli* (EAEC), enterohemorrhagic *E. coli* (EHEC), diffusely adherent *E. coli* (DAEC), and adherent invasive *E. coli* (AIEC) (Mitchell et al., 2015; Singer, 2015; Fratamico et al., 2016; Khasapane et al., 2025a). The ExPEC strains have been identified in sheep and goats; however, research has more commonly focused on their occurrence in poultry, cattle, and pigs (Ajibola et al., 2025; Asmelash et al., 2026). In small ruminants, ExPEC may occur as part of the intestinal microbiota without causing clinical disease and can be transmitted through direct animal contact or the food chain (Fratamico et al., 2016).

With the use of whole genome sequencing (WGS), *E. coli* subtypes can be precisely identified using serotyping and pathotyping based on gene content or allelic variation (Robins-Browne et al., 2016). Additionally, WGS detects single-nucleotide polymorphisms (SNPs) in the core genome, which provides a more accurate method for monitoring the development and dissemination of specific strains compared with traditional approaches (Robins-Browne et al., 2016; Lekota et al., 2024). Genome reduction and horizontal gene transfer (HGT) are the primary drivers of genome size variation in *E. coli*, which ranges from 4.6 to 5.9 Mbp and includes plasmids and prophages (Robins-Browne et al., 2016). New combinations of virulence factors or acquisition from unrelated species can lead to the emergence of novel diarrheagenic *E. coli* (DEC) pathotypes (Feng et al., 1998). Most virulence genes are acquired through mobile genetic elements (MGEs), including plasmids, bacteriophages, pathogenicity islands, and transposons (Leimbach et al., 2013). Rapid gene exchange through conjugation, transduction, or transformation is facilitated by HGT, homologous recombination, and MGEs; in some cases, complete phenotypes can be transferred in a single step (Juhas et al., 2009; Wiedenbeck and Cohan, 2011). In contrast to commensals, which typically have smaller, more streamlined genomes, pathogenic strains often possess larger genomes due to the acquisition of accessory genetic elements (Leimbach et al., 2013).

Additionally, MGEs contribute to the spread of antimicrobial resistance (AMR), a global health concern exacerbated by the misuse of antibiotics in both animals and humans (Leekitcharoenphon et al., 2021). Antibiotic resistance genes are maintained within the intestinal microbiota, including commensal *E. coli*, where selective pressures promote the co-emergence of resistance and virulence traits. These determinants, frequently encoded on transferable elements, complicate control efforts and facilitate the development of multidrug-resistant strains (Leekitcharoenphon et al., 2021).

Despite the recognized importance of livestock production systems as reservoirs of antimicrobial-resistant *E. coli*, limited genomic data are available comparing isolates from contrasting farming systems in South Africa, particularly in small ruminants. Therefore, this study used whole genome sequencing (WGS) to investigate and compare the phylogenetic relationships, resistome, virulome, and mobile genetic elements of *E. coli* isolates obtained from two livestock production systems in the North West Province, South Africa, to improve understanding of strain diversity, antimicrobial resistance dissemination, and potential public health risks associated with these reservoirs.

## 2 Materials and methods

### 2.1 Culture collection of the strains

The present genomic investigation was conducted on archived isolates available in the North-West University bacterial biobank. Although the original surveillance recovered more *E. coli* isolates, only archived cultures that remained viable and had complete metadata were available for whole-genome sequencing. The isolates were recovered from fecal (*n* = 17), and manure (*n* = 2) samples collected from commercial sheep and goat farms in Ventersdorp and Potchefstroom, North West Province, South Africa, during a previous surveillance study of Enterobacteriaceae within livestock production systems (Howard et al., 2026). These isolates were therefore selected for whole-genome sequencing based on confirmed species identification and culture viability following long-term storage.

### 2.2 Antimicrobial susceptibility testing

Kirby-Bauer disk diffusion was used to examine the susceptibility of *E. coli* isolates to various antibiotics. A total of six antibiotic disks from five classes were used. Kirby-Bauer disk diffusion was performed according to Clinical and Laboratory Standards Institute (CLSI) guidelines. A 0.5 McFarland standard bacterial suspension was prepared from pure colonies. *E. coli* isolates were tested against selected antibiotics, namely: Ampicillin (AMP), Erythromycin (E), Gentamycin (CN), Streptomycin (S), Ceftriaxone (CRO) and Meropenem (MEM). The antibiotic disks were purchased from ThermoFischer Scientific™ (ThermoFisher, South Africa). The antibiotic disks were placed on Mueller–Hinton agar (Merck, Germany). Thereafter, plates were incubated in an inverted position at 37 °C for 16–18 h, and zones of inhibition were observed. Based on the zone diameter, antibiotics were assigned to the following categories: susceptible, intermediate, and resistant, according to the Clinical and Laboratory Standards Institute (CLSI) antimicrobial susceptibility testing standards (CLSI 2023). The organism was considered multidrug-resistant (MDR) if it showed resistance to at least three distinct antibiotic classes (Magiorakos et al., 2012; Khasapane et al., 2025b; Motlhaping et al., 2025).

### 2.3 Genomic DNA extraction and whole genome sequencing

Genomic DNA was extracted using the Macherey-Nagel Nucleospin Microbial DNA extraction kit (Qiagen, Germany) according to the manufacturer's protocol. The Qubit 2.0 Fluorometer (Life Technologies, Carlsbad, California, USA) was used for quantifying genomic DNA. Finally, the genomic DNA was stored at −20 °C until further analysis. Whole-genome sequencing was performed at the North-West University Sequencing Facility using the Illumina NextSeq 2000 platform. Genomic DNA was quantified using the Qubit™ dsDNA HS Assay Kit (Thermo Fisher Scientific, USA) to ensure optimal input concentration for library preparation. Sequencing libraries were constructed using the Illumina DNA Prep Kit, which includes enzymatic fragmentation, end repair, A-tailing, adaptor ligation, and amplification with unique dual indices. Fragment size distribution and library quality were assessed on the Qiaxcel (Qiagen, USA). Equimolar pooled libraries were sequenced using paired-end chemistry (2 × 150 bp), generating high-coverage datasets for downstream analysis.

### 2.4 Bioinformatics analysis

***Quality control, assembly and annotation***

Raw sequencing reads were quality-checked using FastQC v0.10.1 (Andrews et al., 2010). High-quality reads were assembled *de novo* using MEGAHIT v1.1.2 (Li et al., 2015) with a multi-k-mer strategy (*k* = 21, 33, 55, 77, 99, and 127) and a minimum contig length of 500 bp. Assembly quality metrics, including N50, total length, and GC content, were calculated using QUAST v2.3 (Gurevich et al., 2013; de Wet et al., 2025;), while genome completeness and contamination were assessed using CheckM v1.1.6 (Parks et al., 2015). Genome annotation and statistical profiling were performed using Anvi'o v7.1 (Eren et al., 2015), with gene prediction carried out using Prodigal v2.6.3 (Hyatt et al., 2010). Predicted coding sequences (CDSs) were clustered using the Markov Cluster Algorithm (MCL) based on BLASTp similarity, and RNA genes were annotated using tRNAscan-SE (Dongen, 2000; Chan et al., 2021).

#### 2.4.1 In silico identification and phylogenetic reconstruction

Species identification and multilocus sequence typing (MLST) of *E. coli* isolates were performed using PubMLST (https://pubmlst.org, accessed May 22, 2025). Isolates that could not be matched to existing profiles in PubMLST were considered potentially novel allelic combinations and were submitted to Pasteur MLST schemes for curation and assignment of new STs (Heljanko et al., 2024a) and subsequently submitted to PubMLST. Average nucleotide identity (ANI) was calculated using the anvi-compute-genome-similarity function in Anvi'o v8.0 (Eren et al., 2015), which incorporates the PyANI software package (Pritchard et al., 2026) for pairwise genome comparisons. *In silico* serotyping was performed with EcTyper v2.0.2 (Bessonov et al., 2021)., and phylogroup classification was conducted with ClermonTyping v1.4.0 using the updated Clermont scheme (Beghain et al., 2018). Whole-genome single-nucleotide polymorphisms (SNPs) were identified using Snippy v4.6.0 (Seemann, 2015). Recombinant regions were removed using Gubbins v3.2.1 (Croucher et al., 2015), and invariant sites were extracted with SNP-sites v2.5.1 (Page et al., 2016). The resulting alignment was used for phylogenetic inference. Maximum-likelihood phylogenies were constructed using IQ-TREE v2.2.0.3 (Minh et al., 2020), with 1,000 ultrafast bootstrap replicates and model selection performed using ModelFinder. Pairwise SNP distance matrices were generated using snp-dists v0.8.2 (Seemann et al., 2018) and visualized in RStudio v4.3.1 (R Core Team, 2023) using the pheatmap v1.0.12 package.

### 2.5 Resistome, virulome, and mobilome profiles analysis

The resistome, virulome, and mobilome profiles of each genome were characterized using ABRicate v1.0.1 (Vieira et al., 2023; Sizana et al., 2026). Resistance genes were screened against the ResFinder database (Vieira et al., 2023), virulence-associated genes were identified using the Virulence Factor Database (VFDB) (Chen et al., 2005; Liu et al., 2019; Motlhaping et al., 2026), and plasmid replicons were detected using PlasmidFinder (Carattoli et al., 2014). A minimum identity and coverage threshold of 70% (–minid 70; –mincov 70) was applied across all databases. Plasmid classification was further confirmed using MOB-suite v3.1.2 (Robertson and Nash, 2018), and mobile genetic elements were identified using IslandViewer4, which integrates IslandPick, SIGI-HMM, and IslandPath-DIMOB methods (Bertelli et al., 2017). All genomes were compared against the complete reference genome of *Escherichia coli* ATCC 8739.

## 3 Results

### 3.1 Average nucleotide identity analysis

Average nucleotide identity (ANI) analysis showed that all 19 isolates shared pairwise ANI values of approximately 97.1%-100%, confirming that they belong to the species *E. coli* (Figure 1). Hierarchical clustering separated the isolates into two major genomic clusters, each characterized by high within-cluster ANI (>99%), indicative of closely related lineages. Several isolate pairs, including Ec_S181A and Ec_S181B, exhibited almost identical genomes (ANI of 99.99%), suggesting a common genetic background. In contrast, Ec_GRF5 and, to a lesser extent, Ec_GXF14 formed distinct branches with lower ANI values (approximately 97–98%), indicating greater genomic divergence while remaining well above the accepted species delineation threshold. Overall, the ANI analysis demonstrates considerable intraspecies genomic diversity among *E. coli* isolates recovered from sheep and goat farming systems, with evidence for multiple co-circulating lineages rather than a single dominant clone.

**Figure 1:**
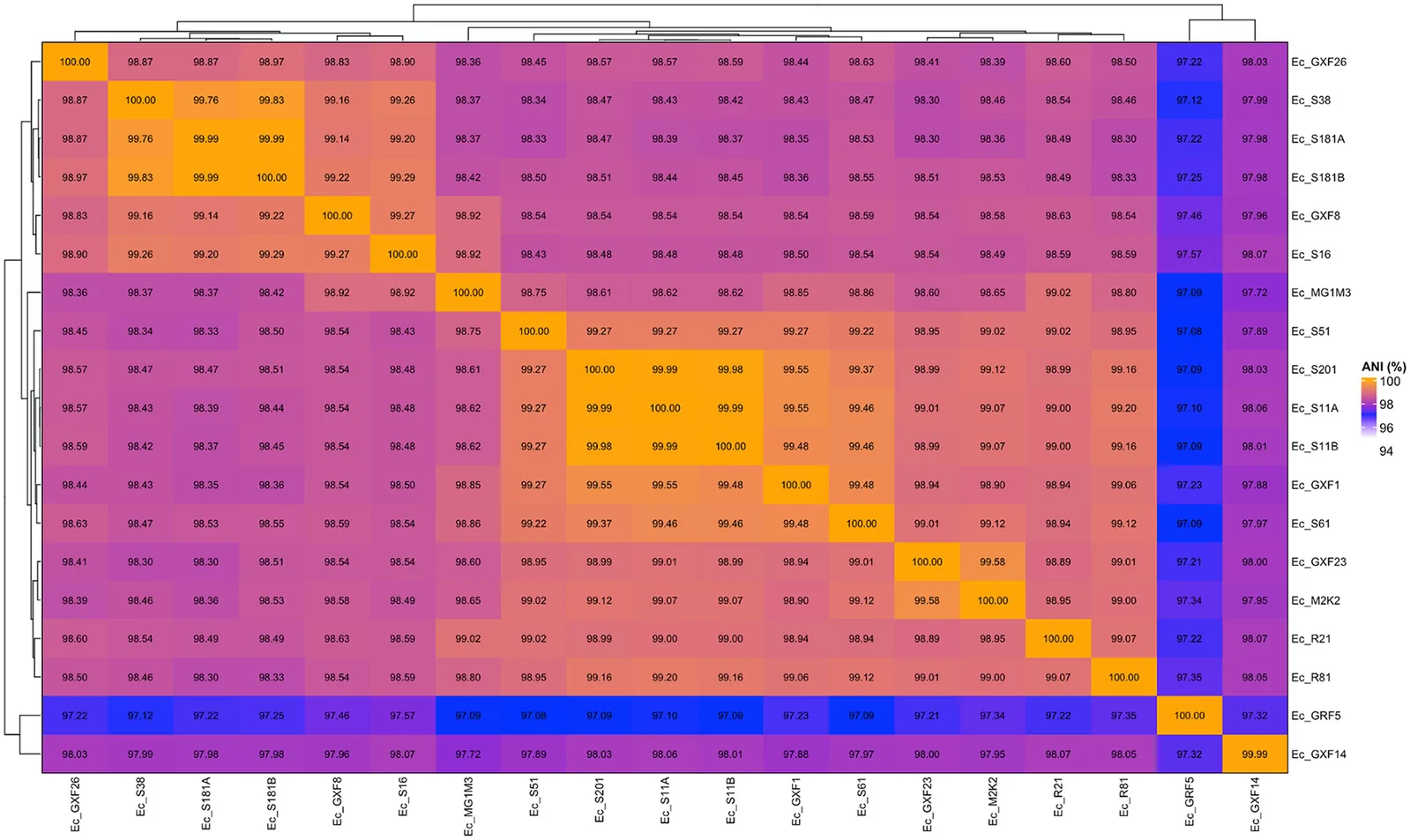
Heatmap of the 19 *E. coli*, indicating the core and accessory genes as well as the average nucleotide identity placement of the sequenced genomes highlighted in blue. The heatmap confirms species identity (97.1–100% ANI) and reveals two major genomic clusters with evidence of multiple co-circulating lineages rather than a single dominant clone. The presence/absence of gene clusters orders the central dendrogram clustering of samples, ordered using D, Euclidean; L, Ward.

### 3.2 Genomic metrics and multilocus sequence typing as well as serotyping

A total of 19 isolates were recovered from both fecal (*n* = 17) and manure (*n* = 2) samples and subjected to whole-genome sequencing. Among the sequenced genomes, two and seventeen were collected from samples obtained in Matlwang and Ventersdorp, respectively. The genome sizes of the 19 *E. coli* isolates ranged from 4.7 to 5.4 Mb, with an average GC content of 50.8%, consistent with values typically reported for *E. coli*. The number of contigs among the assembled genomes ranged from 50 to 2,782. The number of CDS ranged from 4,132 to 7,680. Using multiple multilocus sequence typing (MLST) schemes, all isolates were typed as *E. coli* (Table 1). Of the 19 genomes, four were assigned to sequence type ST10, three to ST155, five were unassigned or represented novel sequence types, and the remaining isolates were distributed among various other known STs. Strains with novel sequence types were assigned new STs, namely: strain Ec_GXF8 as ST3675 or STc669; Ec_GXF14 as ST6fd1 or STc4d4; Ec_S16 as ST2627 or ST3313; Ec_R8-1 as ST2903; and Ec_GXF23 as ST4ed1 or ST7e3b.

**Table 1:** Genomic features of 19 assembled *E. coli* isolates sequenced in this study.

Sample	Isolation source	Location	Number of contigs	Genome size (Mb)	GC content (%)	# of rRNA	# of tRNA	CDS	N50	Sequence type (STs)	Serotype
**Ec_R8-1**	Sheep feces	Ventersdorp	122	5.3	52	11	82	4,511	110775	^*^Untypable	H4
**Ec_GRF5**	Goat feces	Ventersdorp	118	5.4	50.5	6	86	4,882	150921	362	015:H1
**Ec_GXF23**	Goat feces	Ventersdorp	2,782	4.7	51	14	121	7,680	25805	^*^Untypable	H26
**Ec_GXF26**	Goat feces	Ventersdorp	67	5.0	50.5	5	82	4,606	177521	23	O8:H9
**Ec_M2K2**	Manure	Ventersdorp	71	4.9	51	7	81	4,539	149434	189	O131:H26
**Ec_MG1M3**	Manure	Ventersdorp	389	5.3	50.5	9	84	4,569	79684	398	O25-
**Ec_R2-1**	Sheep feces	Ventersdorp	2,546	4.8	50.5	10	115	7,336	11672	361	Unknown
**Ec_S1_1A**	Sheep feces	Ventersdorp	123	4.9	51	8	82	4,562	113628	10	O185:H12
**Ec_S1_1B**	Sheep feces	Ventersdorp	129	4.9	51	7	82	4,523	106338	10	O185:H12
**Ec_S5-1**	Sheep feces	Ventersdorp	69	4.8	50.5	10	83	4,340	156958	215	O8:H32
**Ec_S6_1**	Sheep feces	Ventersdorp	156	4.8	51	6	78	4,422	81992	1415	O176:H34
**Ec_S16**	Sheep feces	Matlwang	1,965	4.8	51	3	54	4,132	2604	^*^Untypable	Unknown
**Ec_S18_1A**	Sheep feces	Ventersdorp	98	5.1	50.5	7	82	4,690	172480	155	O107/O117:H20
**Ec_S20-1**	Sheep feces	Ventersdorp	120	4.8	51	9	84	4,489	112528	10	O185:H12
**Ec_S38**	Sheep feces	Matlwang	131	5.0	50.5	5	79	4,629	119887	155	O64:H20
**Ec_S18_1B**	Sheep feces	Ventersdorp	50	4.6	51	3	71	4,296	185343	155	O117/O107:H20
**Ec_GXF14**	Goat feces	Ventersdorp	87	4.6	50.5	3	76	4,248	86141	^*^Untypable	H45
**Ec_GXF8**	Goat feces	Ventersdorp	1,745	5.0	50.5	5	78	4,812	3960	^*^Untypable	O160:H11
**Ec_GXF1**	Goat feces	Ventersdorp	141	4.9	50.5	11	88	4,660	100292	10	O71:H48

^*^Strains that were assigned a new sequence type.

*In silico* serotyping revealed clonal groups (O185:H12 and O107/O117:H20) that occurred repeatedly among sheep-derived isolates, whereas manure samples harbored more diverse serotypes (Table 1). Three sheep-derived isolates from Ventersdorp (Ec_S1_1A, Ec_S1_1B, and Ec_S20-1) were consistently typed as O185:H12, forming a distinct serogroup cluster. Similarly, two isolates (Ec_S18_1A and Ec_S18_1B) were predicted as O107/O117:H20, consistent with close relatedness and possible minor antigenic variation within the lineage. Several isolates exhibited distinct serotypes, including O64:H20 in strain Ec_S38 from Matlwang, O8:H32 in strain Ec_S5-1 from Ventersdorp, O15:H1 in strain Ec_GRF5 from Ventersdorp, and O131:H26 in strain Ec_M2K2 from manure in Ventersdorp. The manure-derived isolate Ec_MG1M3 was typed as O25:-, lacking a detectable H antigen, while strain Ec_R8-1, isolated from sheep feces in Ventersdorp, was identified as H4 only. The H26 antigen was identified in strain Ec_GXF23 from goat feces in Ventersdorp, with no corresponding O-type assignment. One sheep isolate from Matlwang, Ec_S16, remained untyped, with no O or H antigen detected.

### 3.3 Virulence gene profiles examined across the *Escherichia coli* isolates

A total of 95 distinct virulence-associated genes were identified across the 19 *E. coli* isolates analyzed in this study (Figure 2). These genes were classified into six functional categories: adhesins and fimbriae, iron acquisition systems, toxins, type II secretion system (T2SS) components, outer membrane/surface proteins, and effector/regulatory elements. The core virulome was defined as genes present in at least 70% of isolates (≥ 14/19); 23 genes met this criterion. These included genes encoding type III secretion system (T3SS) effector proteins (*espX5, espX4, espL1, espR1*), curli biosynthesis proteins (*csgB, csgD, csgF, csgG*), and components of enterobactin-mediated iron acquisition (*entA, entB, entC, entD, entE, entF, entS, fepA, fepB, fepC, fepD, fepG, fes*). In addition, *ecpE* (*E. coli* common pilus, involved in adhesion and biofilm formation) and *ompA* (membrane integrity, immune evasion, and adhesion) were present in over 70% of isolates. Virulence gene content nonetheless varied considerably across isolates. Strains Ec_GRF5 and Ec_GXF26 carried the largest virulence gene repertoires, each with 50–70 genes spanning nearly all functional categories, including rare or strain-specific adhesins (*afaA-VIII, afaB-VIII, f17d-C, fdeC, iucA, iucB*) and effector proteins (*espX5, espY4*). The *astA* gene, encoding the enteroaggregative heat-stable enterotoxin 1 (EAST-1), was detected in six isolates (Ec_GRF5, Ec_S20-1, Ec_S1_1A, Ec_S1_1B, Ec_S16, and Ec_MG1M3). By contrast, isolates Ec_MG1M3 and Ec_S16 harbored a reduced virulence gene repertoire (16–32 genes), consisting predominantly of conserved core virulence-associated genes. Several isolates, including Ec_R8-1, Ec_GXF23, Ec_M2K2, Ec_S5-1, and Ec_GXF1, carried genes associated with adhesion, iron acquisition, and serum survival. Similarly, isolates Ec_GRF5, Ec_S38, Ec_S18_1A, Ec_S18_1B, and Ec_GXF26 possessed a broader repertoire of virulence-associated genes, including those involved in adherence, toxin production, secretion systems, and iron uptake. Strain Ec_GXF8 carried the *fimH* gene encoding type 1 fimbriae, together with the *pap* gene cluster and *sfa* adhesin genes. Several virulence genes were rare, occurring in only one or two isolates. The *papX* gene (mobility inhibitor) was detected only in strain Ec_GRF5. The *gtrA* gene (O-antigen modification) was detected only in strain Ec_GXF23. The *espY1* gene (encoding a T3SS effector protein) was detected in strains Ec_S5-1 and Ec_GXF1. The *afaD-VIII* gene (host invasion-associated protein) was detected in strain Ec_GRF5. Genes constituting the T2SS operon (*gspC* through *gspM*) were inconsistently present, being detected in isolates Ec_S6_1, Ec_S38, Ec_GRF5, Ec_M2K2, Ec_R2-1, Ec_S18_1A, Ec_S18_1B, Ec_GXF26, and Ec_R8-1.

**Figure 2:**
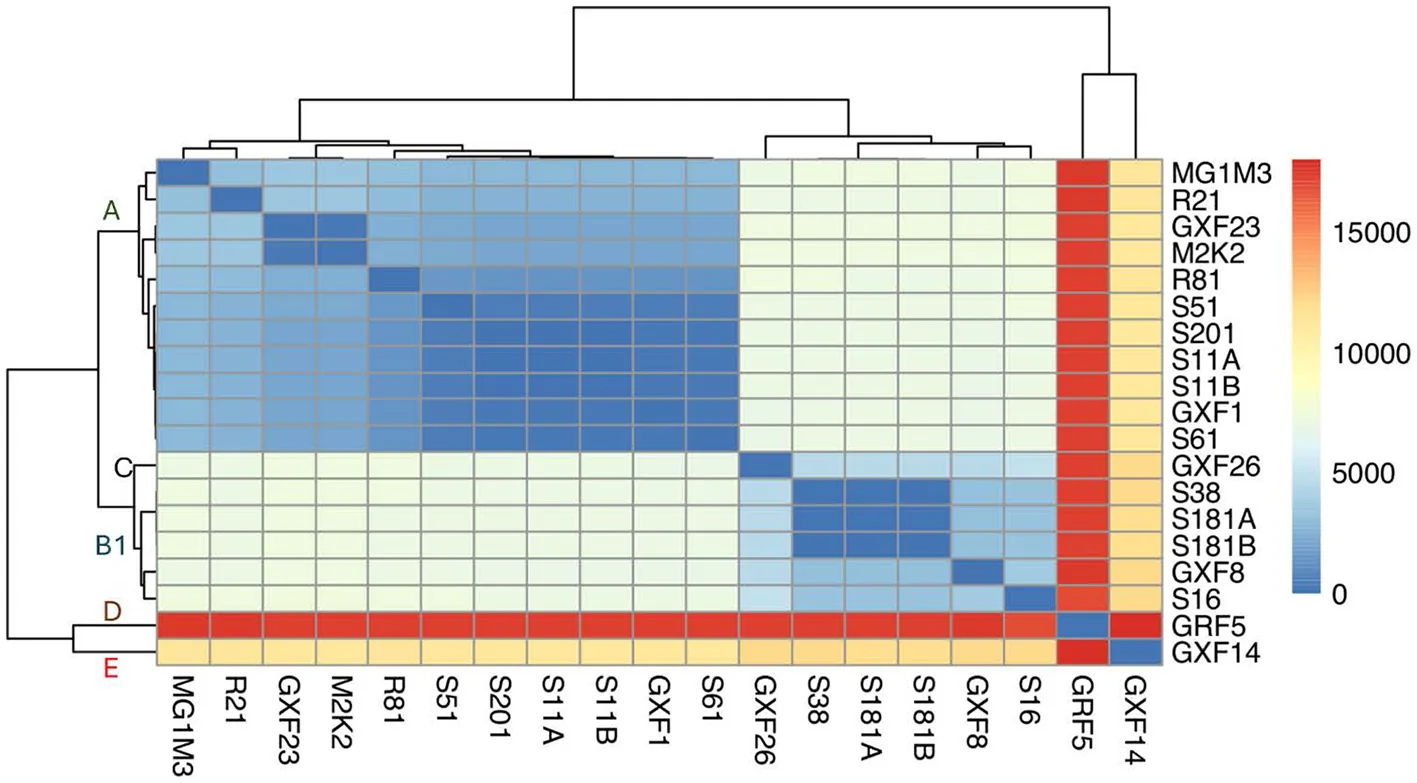
Whole genome single-nucleotide polymorphism tree based on 19 *E. coli* and phylogroups placement. A total of 95 virulence genes across six functional categories were identified; 23 genes formed the core virulome, while gene content varied widely between individual isolates. The phylogenetic tree is based on the maximum likelihood method, generated from constant SNPs across the compared genomes. Heat map indicates constant SNP distance similarities (blue) to delineation (red) based on distance matrix.

### 3.4 Virulence–phylogroup associations

Based on whole-genome SNP and phylogroup analysis, the 19 *E. coli* isolates were assigned to five phylogroups: A (11 isolates), B1 (4), C (1), D (1), and E (1) (Figure 3). Phylogroups A and B1 accounted for approximately 89% of the isolates, while phylogroups C, D, and E were each represented by a single isolate. Most sheep-derived isolates clustered within phylogroups A and B1, whereas goat-derived isolates were distributed across phylogroups A, C, D, and E. The distribution of virulence-associated genes varied among phylogroups. The *astA* gene was detected in six isolates belonging to phylogroup A (Ec_GRF5, Ec_S20-1, Ec_S18_1A, Ec_S18_1B, Ec_S16, and Ec_MG1M3). Isolates Ec_S16 (phylogroup B1) and Ec_MG1M3 (phylogroup A) possessed comparatively fewer virulence-associated genes than the remaining isolates. Strains Ec_GRF5 (phylogroup D) and Ec_GXF14 (phylogroup E) carried genes including *afaD-VIII, papX*, and components of the type II secretion system (T2SS). Isolates Ec_GXF26 (phylogroup C), Ec_S18_1A, and Ec_S18_1B carried the *espY1* gene, while strain Ec_GXF8 (phylogroup A) harbored *fimH*, the *pap* gene cluster, and *sfa* adhesin genes. In addition, genes such as *gtrA* (Ec_GXF23), *papX* (Ec_GRF5), and several T2SS-associated genes were detected in only a subset of isolates, highlighting genomic variability in the distribution of virulence-associated determinants across the collection.

**Figure 3:**
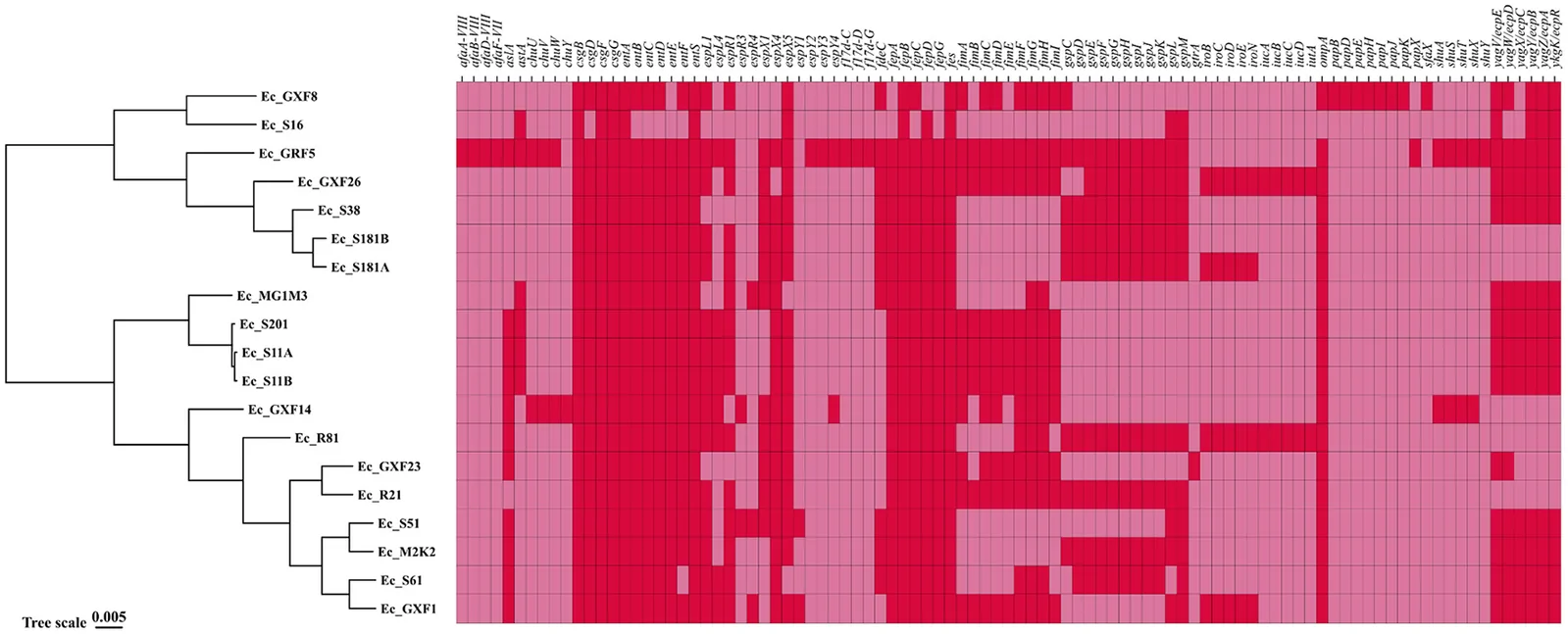
Comparative analysis of the virulence gene profiles found within the 19 *E. coli* genomes isolated from feces and manure in sheep and goats. Phylogenetic analysis assigned isolates to five phylogroups (A, B1, C, D, E), with A and B1 predominating (89%). Virulence-associated gene content was heterogenous across isolates, with no consistent pattern linked to phylogroup. Dark pink – present, Light pink – absent. The phylogenetic tree clusters the isolates based on average nucleotide identity (ANI). The Ec represents *Escherichia coli* in the strain names.

### 3.5 Antibiotic susceptibility profiles of the *E. coli* strains

All *E. coli* isolates were susceptible to meropenem and ceftriaxone (carbapenem and cephalosporin classes, respectively) (Supplementary Figure 1). Eighteen of 19 isolates were susceptible to gentamicin, with Ec_S18_1B being the exception. All isolates except Ec_S16 were resistant to ampicillin. Four isolates showed intermediate resistance to erythromycin (Ec_GRF5, Ec_GXF8, Ec_M2K2, and Ec_S5-1), and four isolates showed intermediate resistance to streptomycin (Ec_S6_1, Ec_R2-1, Ec_MG1M3, and Ec_GXF23). Overall, 95% (*n* = 18) of isolates were resistant to ampicillin, 74% (*n* = 14) were resistant to erythromycin, and 32% (*n* = 6) were resistant to streptomycin (Supplementary Table 1).

*Escherichia coli* isolates resistant to three or more antibiotic classes were classified as multidrug-resistant (MDR). Of the 19 isolates, five (26.3%) met this criterion: Ec_S18_1B, Ec_GXF14, Ec_GXF1, and Ec_R8-1 (all from Ventersdorp), and Ec_S38 (from Matlwang). These five isolates showed resistance to three antibiotic classes: β-lactams (ampicillin), aminoglycosides (streptomycin and gentamicin), and macrolides (erythromycin).

### 3.6 Antimicrobial resistance genes examined across *Escherichia coli* genomes

A total of 24 distinct antibiotic resistance genes (ARGs) were identified across the 19 *E. coli* genomes (Figure 4), encoding mechanisms including multidrug efflux transporters, tetracycline efflux pumps, and β-lactamase enzymes, and conferring resistance to tetracyclines, β-lactams, chloramphenicol, erythromycin, and various disinfectants/antiseptics. The multidrug efflux pump gene *mdf (A)_1* was the most frequently detected, found in 18 of 19 isolates (94.7%), followed by the tetracycline resistance gene *tet(A)_6* and the β-lactamase gene *bla*_TEM − 1B_1_, each present in 11 isolates (57.9%). Several ARGs were detected in only one isolate each (5.3%), including *aph3*′*)-Ia_3* and *aac(3)-IId_1* (aminoglycoside-modifying enzymes), *fosA3_1* (fosfomycin resistance via glutathione transferase), *bla*_CTX − M−14_1_ (β-lactamase), *qnrS1_1* (quinolone resistance), and *catA1_1* (chloramphenicol acetyltransferase). Notably, *fosA3_1* and *bla*_CTX − M−14_1_ co-occurred in the same isolate, strain Ec_GXF23, where the two genes are present on a single plasmid, linking an ESBL determinant and a plasmid-mediated fosfomycin-resistance determinant within a single mobile element.

**Figure 4:**
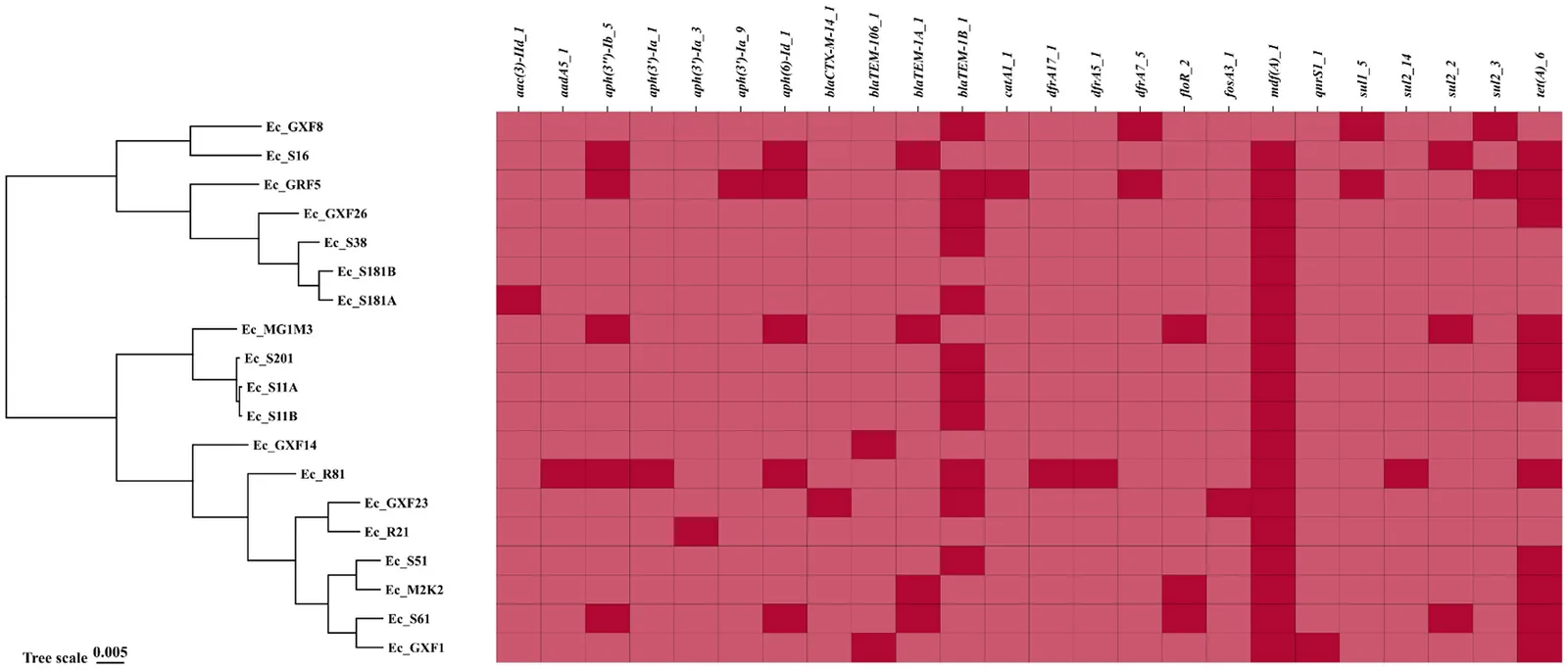
Antibiotic resistance gene profiles determined among the 19 *E. coli* genomes using the ResFinder database. The phylogenetic tree clusters the isolates based on average nucleotide identity. A total of 24 distinct ARGs were identified, with *mdf****(A)****_1* the most prevalent (94.7%). Dark pink – present, Light pink – absent.

Isolates Ec_GRF5 (goat) and Ec_R8-1 (sheep), both from Ventersdorp, carried the largest ARG complements (10 genes each), conferring resistance to a broad range of antibiotic classes. Both isolates carried sulfamethoxazole resistance genes (*sul1_5, sul2_3, sul2_14*) and trimethoprim resistance genes (*dfrA7_5, dfrA17_1, dfrA5_1*), together with *tet(A)_6* and *blaTEM-1B_1* (conferring resistance to ampicillin, amoxicillin, and cephalothin). Both isolates also shared *aph(6)-Id_1, aph3*′'*)-Ib_5*, and *aph3*′*)-Ia_1* (neomycin, kanamycin, and streptomycin resistance). Ec_GRF5 additionally carried *catA1_1* (chloramphenicol resistance), while Ec_R8-1 additionally carried *aadA5_1* (streptomycin resistance). Isolates Ec_MG1M3 (manure) and Ec_S6_1 (sheep, Ventersdorp) each carried seven ARGs spanning aminoglycoside, β-lactam, tetracycline, florfenicol, and sulfamethoxazole resistance, plus a multidrug efflux pump gene; Ec_S16 (sheep, Matlwang) carried six ARGs. Notably, the genotypic and phenotypic resistance profiles of these three isolates did not fully align. Phenotypically, all three isolates were resistant only to ampicillin and erythromycin, yet none carried a macrolide resistance gene in their genomic ARG profile. This genotype-phenotype discrepancy may reflect resistance mechanisms not captured by the ARG database used such as efflux-mediated or mutation-mediated macrolide resistance. By contrast, five isolates carried the fewest ARGs (one to two genes each): Ec_R2-1, Ec_S18_1B, Ec_S38, and Ec_S1_1B (sheep), and Ec_GXF14 (goat). All five carried genes encoding β-lactamase and multidrug efflux pump functions, except for Ec_R2-1, which instead carried *aac(3)-IId_3* (aminoglycoside resistance). This pattern indicates a heterogeneous distribution of resistance determinants among isolates from different hosts and sources.

### 3.7 Genomic attributes of plasmids identified in the 19 *Escherichia coli* strains

A total of 71 plasmids were identified across the 19 *E. coli* strains (Supplementary Table 2), with varied mobility profiles. Thirteen plasmids (AA175, AA372, AD446, AD672, AF232, AG294, AH321, AA374, AA176, AA474, AB193, AA974, and AC770) were each present in multiple genomes, collectively spanning 15 of the 19 strains; these strains were isolated from nine sheep-derived fecal samples, five additional fecal samples, and one manure sample. Strain Ec_GXF8 carried the highest number of plasmids of any isolate (*n* = 12), while strains Ec_GXF14, Ec_S6_1, and Ec_S18_1B each carried only a single plasmid. Despite its high plasmid load, only two of Ec_GX8′s 12 plasmids carried ARGs, and both were non-mobilizable, suggesting limited potential for horizontal transfer of resistance from this strain via plasmids.

Of the 71 plasmids, 17 were classified as conjugative, 19 as mobilizable, and 35 as non-mobilizable; overall, 22 of 71 plasmids (31%) carried at least one ARG. Among conjugative plasmids, nine of 17 lacked ARGs. Plasmid AA372 was identified in both Ec_S18_1A (sheep) and Ec_GXF8 (goat), both from Ventersdorp; both strains carried the plasmid, which was conjugative but carried no ARGs. Plasmids AA176 and AA303, found respectively in Ec_R8-1 (sheep) and Ec_GRF5 (goat), also from Ventersdorp, carried the highest ARG counts among conjugative plasmids (five and seven genes, respectively), including *sul1_5, dfrA7_5, catA1_1*, *bla*_TEM − 1B_1_, *aph(6)-Id_1, aph3*′‘*)-Ib_5, sul2_3, sul2_14*, and *aph3*′*)-Ia_1*. Plasmid AA474, detected in three strains (Ec_GXF26, Ec_S16, and Ec_R8-1), was consistently conjugative, though ARGs (*dfrA17_1, aadA5_1, tet(A)_6*, *bla*_TEM − 1B_1_) were present only in Ec_GXF26 and Ec_R8-1. Among ARG-carrying plasmids overall, conjugative plasmids carried between 1 and 7 ARGs, mobilizable plasmids between 1 and 6 ARGs, and non-mobilizable plasmids between 1 and 6 ARGs (ranges refer to ARG counts per individual plasmid, not plasmid counts). Plasmid AB193, found in strain Ec_S6_1 (sheep, Ventersdorp), carried the most ARGs of any single plasmid (*n* = 6): *floR_2, tet(A)_6, aph(6)-Id_1, aph3*′'*)-Ib_5, sul2_2*, and *bla*_TEM − 1A_; this plasmid was conjugative.

Most plasmids had GC content of approximately 50–51%, similar to that of their host strains' chromosomal genomes. Eighteen plasmids deviated from this range by more than 5%, including: AF190 (Ec_S16), AE974 (Ec_R8-1), AD484 (Ec_GXF26), AC621 (Ec_MG1M3), AC305 (Ec_R2-1), AC770 (Ec_S1_1A), AD672 (Ec_S1_1B and Ec_S5-1), AB193 (Ec_S6_1), AA179/AA372/AF232 (Ec_S18_1A), AB947/AD690 (Ec_S38), and AC082/AD672/AG271/AH321 (Ec_GXF8). Ten of the 71 plasmids encoded tRNA genes, with copy numbers ranging from 1 to 4. Plasmid ACG21, from strain Ec_MG1M3, was the only tRNA-carrying plasmid with a markedly lower GC content (44.49%) and a relatively high CDS count (*n* = 69). Plasmid AA303 (138 kbp), from strain Ec_GRF5, had the highest CDS count of all plasmids (*n* = 169), one tRNA gene, and a GC content of 50.48%; functional annotation of AA303 identified genes involved in conjugation (*traC, traI, traN, traV* — pilus assembly, DNA transfer, and mating pair stabilization), transposition/resolution (Tn3 transposase, *xerC_3*), plasmid segregation and stability (*parM, herM*), metabolic regulation (*deoR, gntR*), nucleoid occlusion (*noc*), and adhesion (*aidA*), along with numerous hypothetical and putative proteins (Supplementary Figure 2).

A mobilizable IncHI1B-type plasmid, AA374, identified in strain Ec_MG1M3, carried *tet(A)_6, aph(6)-Id_1, aph3*″*)-Ib_5, sul2_2*, and *bla*_TEM − 1A_1_, conferring resistance to tetracyclines, aminoglycosides, sulfonamides, and β-lactams (Supplementary Figure 3). Similarly, strain Ec_S6_1 carried a mobilizable IncR-type plasmid, AB193, with a comparable resistance profile: *floR_2* (chloramphenicol), *tet(A)_6, aph(6)-Id_1, aph3*″*)-Ib_5, sul2_2*, and *bla*_TEM − 1A_1_ (Supplementary Figure 4), indicating multidrug resistance potential mediated through mobilization.

Seven unclassified plasmids were identified across five strains (Ec_MG1M3; Ec_S1_1A and Ec_S1_1B; Ec_S20-1; Ec_S38; and Ec_S18_1B). BLAST comparison against the NCBI database showed that the plasmids from Ec_MG1M3, Ec_S1_1A, Ec_S1_1B, and Ec_S20-1 shared 100% nucleotide identity with *E. coli* strain GF66.1 plasmid pGF66.1-F (accession CP172200); none of these plasmids carried ARGs. A plasmid from Ec_S18_1B shared 100% identity with *E. coli* PSS-08-2 plasmid pPSS-08-2_2 (accession AP027681). An unclassified plasmid from strain Ec_S38-2ae shared 99.98% identity with *E. coli* strain RHB46-C05 plasmid unnamed3 (accession CP098999), and an unclassified plasmid from strain Ec_S38-85c shared 97.83% identity with *E. coli* strain CUPS04 plasmid pCUPS04D (accession CP180698). Of the seven novel plasmids, five were mobilizable, four of which shared the same replicon type (Col[MG828]), CDS count (1), and plasmid size (1.764 kbp), despite GC content ranging from 46.2% to 50.74%. The two non-mobilizable novel plasmids included the largest of the seven (10.752 kbp, in strain Ec_S18_1B), closely related to *E. coli* PSS-08-2 plasmid pPSS-08-2_2; although the reference plasmid was derived from a colistin-resistant, *mcr*-carrying strain, this novel plasmid lacked ARGs but carried several phage-related genes. The two non-mobilizable plasmids had distinct replicon types (rep_cluster_2335 and IncHI1B) and higher CDS counts than the mobilizable unclassified plasmids.

A total of 21 plasmid replicon types were identified across the dataset (Supplementary Figure 5). The most prevalent was po111_1, detected in 10 of the 19 strains, followed by IncFIB(AP001918)_1 (6 strains) and Col(MG828)_1 and IncHI1B(CIT)_1_pNDM-CIT (4 strains each). Plasmid replicons were detected across all three sample sources (sheep, goats, and manure) but were most prevalent among sheep isolates from Ventersdorp. Ten of the 21 replicon types were unique to individual Ventersdorp strains, including IncFII(29)_1_pUTI89 (Ec_GXF1, goat); IncI_Gamma_1 and IncI2_1_Delta (Ec_GXF8, goat); Col440I_1 (Ec_MG1M3, manure); IncN_1 and IncX1_1 (Ec_R2-1, sheep); IncI2_1 and IncFII(pCoo)_1_pCoo (Ec_S18_1A, sheep); and IncFIA_1 and IncFIB(pB171) _1_pB171 (Ec_M2K2, manure).

### 3.8 Mobile genetic elements

A total of 80 mobile genetic element (MGE)-associated genes were identified across the 19 *E. coli* genomes (Figure 5), comprising genes linked to transposons (*n* = 12), integrative and conjugative elements (ICEs, *n* = 18), insertion sequences (*n* = 2), plasmids (*n* = 9), prophages (*n* = 28), and integrons (n = 11). Collectively, these elements represent potential routes for the dissemination of antimicrobial resistance, virulence traits, and genes for metabolic adaptation. Transposon-associated genes *tnpB* (74% of isolates) and *tnpC* (32%) were widely distributed, while *tnpA* was restricted to two Ventersdorp isolates (Ec_M2K2 and Ec_GXF1). Other transposon-related genes, including *rayT, insG*, and *istA*, were detected in multiple isolates.

**Figure 5:**
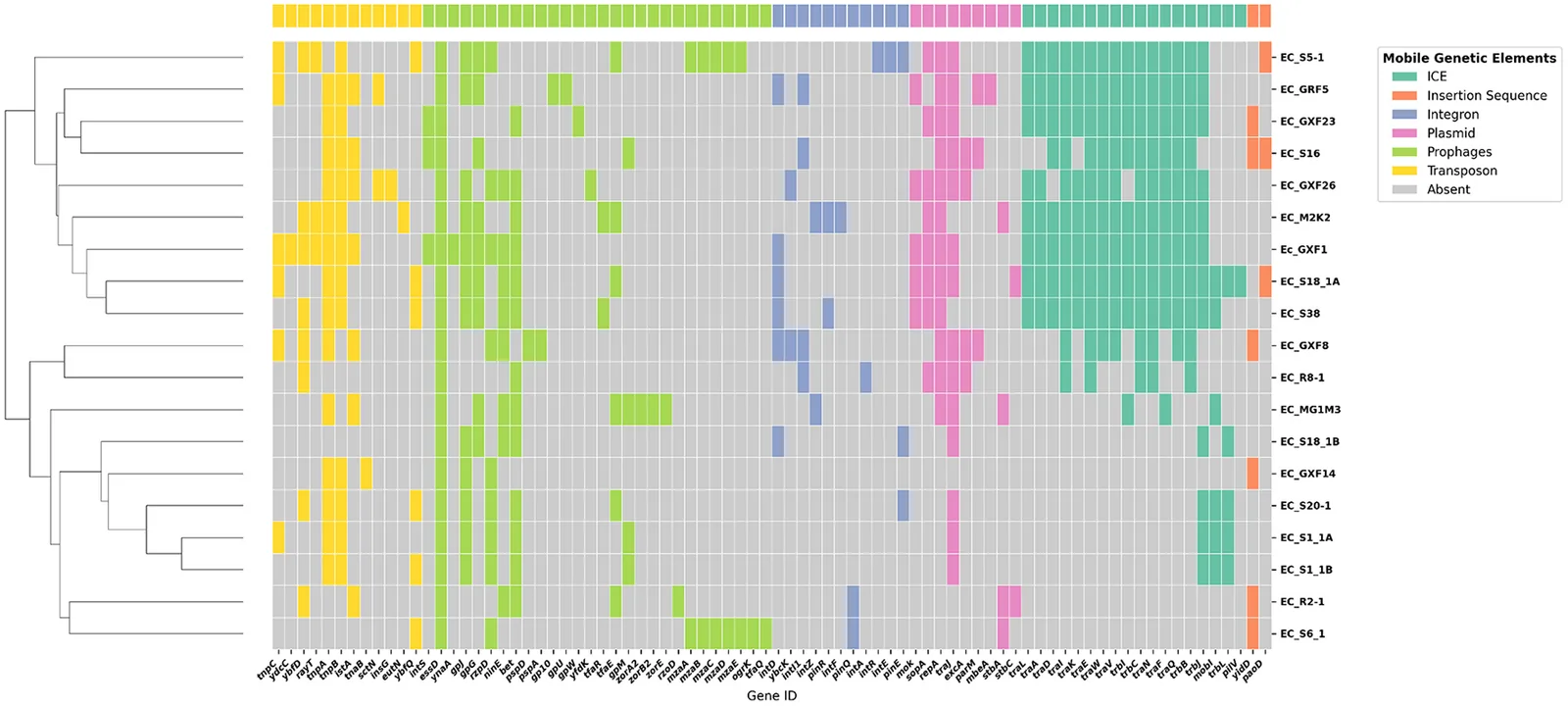
Mobile genetic elements identified among the 19 *E. coli* genomes isolated from small ruminants and manure. A total of 80 mobile genetic element (MGE)-associated genes were identified across the 19 *E. coli* genomes belonging to phages, ICE, transposons, insertion sequences, integrons and plasmids.

ICE-associated genes were the second most abundant MGE group. The conjugation gene *trbL* was the most prevalent (12/19 isolates), particularly among Ventersdorp isolates (Ec_S1_1B, Ec_S1_1A, Ec_S20-1, Ec_S18_1B, and Ec_S18_1A). Additional ICE genes (*traA, traD, traI, traE, traK, traW, traV, traN, traF*, and *traQ*), essential for conjugative transfer, were detected in 42–58% of isolates.

Plasmid-associated genes were widespread and functionally diverse. The replication gene *repA* was found in 63% of isolates. The partitioning genes *sopA* and *parM*, present in strains Ec_GXF8, Ec_GRF5, and Ec_S16, support stable plasmid segregation. The conjugative transfer gene *traI* was detected in 74% of isolates, while *excA* (entry exclusion protein) was detected in strains Ec_R8-1, Ec_GXF8, Ec_GXF26, and Ec_S16. The maintenance genes *stbA* and *stbC* were detected in multiple isolates, consistent with plasmid persistence across the dataset. Insertion sequence elements were comparatively rare, limited to *yidD* (six isolates) and *paoD* (three isolates).

Prophage-associated genes were particularly abundant, encompassing structural, regulatory, and virulence-linked loci. The *essD* gene (a type VI secretion system effector linked to bacterial competition and virulence) was detected in all 19 strains. The *ynaA* gene (stress response and membrane-associated processes during prophage maintenance) was detected specifically in strain Ec_GXF1 (goat, Ventersdorp). Structural genes *gpL* (portal protein, 63% of strains) and *gpG* (head–tail joining protein, 47% of strains) were also prevalent. Five prophage-associated genes (*mrzA*–*mrzE*) were identified in strains Ec_S5-1 and Ec_S6_1 and may contribute to host-cell interaction, lysis, stress response, and phage gene expression. The *intS* gene (site-specific integrase) was detected in strains Ec_GXF1, Ec_S16, and Ec_GXF23. The stress-response genes *pspA* and *pspD*, involved in maintaining cytoplasmic membrane integrity, were both detected in strain Ec_GXF8. The defense-associated genes *zorA2, zorB2*, and *zorE*, part of the ZorAB-E abortive infection system, were detected in manure-derived strain Ec_MG1M3 (Ventersdorp).

Seven integrase genes from distinct integron classes (*intI1, intI2, intA, intF, intD, intR*, and *intE*) were identified, which mediate site-specific recombination of gene cassettes into and out of integrons. Both *intA* and *intR* were detected in strain Ec_S5-1, and *intA* alone in strain Ec_R8-1; all three relevant strains were sheep-derived. The *intD* gene was the most widely distributed of this group, detected in six strains (Ec_GRF5, Ec_GXF1, Ec_S18_1A, Ec_S38, Ec_GXF8, and Ec_S18_1B). The *pinE* gene (regulator of integrase-mediated recombination) was detected in strains Ec_S20-1, Ec_S18_1B, and Ec_S5-1 (all sheep-derived), while the poorly characterized *ybcK* gene, thought to encode a site-specific recombinase or integrase mediating insertion, excision, or inversion of DNA segments was detected in strains Ec_GXF8 and Ec_GXF26.

## 4 Discussion

To our knowledge, this study presents the first whole-genome sequencing-based genomic characterization of *E. coli* from sheep, goats, and manure in South African small-ruminant production systems, The analyses revealed substantial genomic diversity encompassing globally disseminated sequence types, diverse virulence-associated genes, antimicrobial resistance determinants, plasmids, illustrating the extensive genomic plasticity of livestock-associated *E. coli*. Livestock production environments, especially small ruminant systems in low- and middle-income countries, are often overlooked as reservoirs for antimicrobial-resistant and virulent *E. coli* (Ramatla et al., 2024). Despite their close connection to humans and the environment, limited genomic data are available on the diversity, resistome, and mobilome of *E. coli* from these ecosystems, particularly in South Africa and across Africa. Understanding the composition and dynamics of mobile genetic elements (MGEs) is crucial, as these elements serve as the primary vehicles for the horizontal transfer of antimicrobial resistance and virulence traits (Khasapane et al., 2025b), and this understanding underpins efforts to elucidate the evolutionary mechanisms driving bacterial adaptation. Whole-genome sequencing of 19 isolates uncovered substantial heterogeneity in sequence types, serotypes, and phylogroups, with globally disseminated lineages such as ST10 and ST155 co-existing alongside several novel or rarely reported STs, underscoring ongoing microevolution within small ruminant populations. These findings emphasize the adaptability and genetic flexibility of livestock-associated *E. coli*, indicating that small ruminant systems may serve as reservoirs for antimicrobial resistance and virulence traits within farming environments.

We observed remarkable genomic heterogeneity among isolates, spanning globally distributed sequence types such as ST10 and ST155, as well as several novel or rarely reported STs (e.g., ST3675, ST6fd1, ST2627, ST2903, ST4ed1). ST10 was identified in strains Ec_S1_1A, Ec_S1_1B, and Ec_S20-1, all collected from sheep in Ventersdorp, while ST155 was found in strains Ec_S18_1A, Ec_S18_1B, and Ec_S38, collected from sheep in both Ventersdorp and Matlwang; notably, strains sharing these sequence types also belonged to the same clonal complex. ST10 has previously been reported in *E. coli* isolates from food-producing animals, including small ruminants, in South Africa, where it was identified as one of the most prevalent sequence types among 35 strains isolated from animal specimens, meat, and meat products (Malesa et al., 2024). ST155 has been described elsewhere as dominant in poultry and livestock across sub- Saharan Africa (Foster-Nyarko et al., 2021). However, it has only been indirectly identified in South Africa via wastewater and environmental samples taken in proximity to livestock farms (Heljanko et al., 2024b). No prior reports describe its direct circulation in small ruminants from South Africa. The detection of both ST10 and ST155 in South African sheep therefore provides an important update to existing knowledge, and this study represents, to our knowledge, the first report of ST155 in small ruminants, underscoring the need for ongoing genomic monitoring to protect public and animal health. The detection of several novel or rarely reported STs among isolates from small ruminants further indicates high genomic diversity and ongoing evolution within these *E. coli* populations.

The integration of *in silico* serotyping, ANI clustering, and MLST profiling provided complementary insights into the genetic relationships among the 19 *E. coli* isolates. Isolates sharing identical serotypes were also highly similar in their ANI values and MLST assignments, reflecting clonal expansion at the farm level. For example, the O185:H12 lineage (Ec_S1_1A, Ec_S1_1B, and Ec_S20-1) displayed ANI ≥99.5% and was assigned to ST10, confirming its clonal nature within Ventersdorp flocks; clonal complexes represent assemblages of closely related sequence types identified through phylogenetic analysis of their genetic sequences (Schmidt and Hensel, 2004; Hanage et al., 2005). Likewise, Ec_S18_1A and Ec_S18_1B (O107/O117:H20) clustered with Ec_S38 (O64:H20) at ANI >95% and belonged to ST155, illustrating intra-lineage diversity with antigenic variation alongside strong core-genome relatedness. These findings demonstrate that serotype identity and MLST often converge with ANI signals to delineate clonal complexes (Bessonov et al., 2021), as observed in the ST10–O185:H12 and ST155–O107/O117:H20/O64:H20 associations.

The observed phylogenetic distribution mirrors patterns previously reported in South African livestock-associated strains, where phylogroups A and B1 predominate and typically represent commensal or environmental lineages adapted to animal hosts. Similar trends were noted by Malesa et al. (2024), who found that *E. coli* from ruminants and small livestock on rural South African farms primarily belong to these phylogroups, reflecting ecological adaptation to the gut environment and limited exposure to high antibiotic selection pressure. Pathogenic lineages belonging to phylogroups D and E, which are notable for their association with virulence and potential public health risk, were less common than the commensal groups A and B1 and were found exclusively among goat-derived isolates in this study. Recent genomic surveys of *E. coli* from water, fresh produce, and animal products in South Africa similarly confirm the presence of phylogroup D, albeit at lower frequencies relative to A, B1, and E—for example, phylogroup D represented 6.56% of isolates from water and fresh produce (Richter et al., 2024).

Despite this diversity, a core set of 23 genes was identified as present in at least 70% of strains, including those involved in type III secretion system effectors (*espX5, espX4, espL1, espR1*), curli biosynthesis (*csg* cluster), enterobactin-mediated iron acquisition (*ent* and *fep* clusters, and *fes*), and adhesion/membrane stability (*ecpE, ompA*). This shared virulence backbone reflects conserved mechanisms of adhesion, iron acquisition, and immune evasion that collectively support *E. coli* persistence across diverse hosts and environments. Beyond this core, isolates displayed substantial variability in virulence gene content. Strains such as Ec_GRF5 and Ec_GXF26 carried extensive repertoires of 50–70 genes spanning nearly all functional categories, including rare adhesins (*afaA-VIII, afaB-VIII, f17d-C, fdeC*) and unique effectors (*espY4*), pointing to potentially elevated pathogenic capacity consistent with EHEC or EPEC pathotypes (Johnson et al., 2003; Malberg Tetzschner et al., 2020). EHEC/EPEC-associated loci such as *espL, espR, espX*, and *espY* were consistently identified in strains Ec_GRF5, Ec_S38, Ec_S18_1A, Ec_S18_1B, and Ec_GXF26; such genes are known to facilitate bacterial adherence and host-cell invasion, promote biofilm formation, and enhance survival and persistence by aiding immune evasion (Emody et al., 2003).

By contrast, strains such as Ec_MG1M3 and Ec_S16 exhibited reduced gene repertoires (16–32 genes), largely limited to the core virulome, suggesting lower virulence potential—although functional pathogenicity studies would be needed to confirm this. Beyond the EHEC/EPEC markers, several strains also carried determinants associated with ExPEC and UPEC (urosepsis-associated *E. coli*), including *papAH/papC, sfa/focDE, afa/draBC*, and *iutA*; isolates Ec_R8-1, Ec_GXF23, Ec_M2K2, Ec_S5-1, and Ec_GXF1 met the ExPEC classification criteria proposed by Johnson et al. (2003), which require two or more ExPEC markers. ExPEC and UPEC lineages remain poorly documented in South African sheep and goats: while ExPEC serotypes have been extensively studied elsewhere, and various *E. coli* pathotypes have been examined in South African small ruminants, direct studies on ExPEC distribution in these animals within South Africa are lacking (Ramatla et al., 2024), pointing to a potential and currently under-characterized role for these strains in extraintestinal infections. These findings reinforce the genomic diversity of *E. coli*, indicating that strains from the same source can carry virulence factors associated with markedly different pathotypes a consideration of particular importance when UPEC enters the food chain, where it can facilitate the transmission of virulence and resistance genes to humans, potentially contributing to outbreaks of severe extraintestinal infection and complicating public health interventions (Sarowska et al., 2019).

Additional genes were identified as rare or strain-specific, further illustrating the genomic plasticity of *E. coli*: *papX* (a mobility inhibitor) was found only in Ec_GRF5, *gtrA* (O-antigen modification) was exclusive to Ec_GXF23, *espY1* was detected solely in Ec_S5-1 and Ec_GXF1, and *afaD-VIII* (an invasion-like protein) was unique to Ec_GRF5. The inconsistent distribution of T2SS genes across isolates may likewise reflect differences in protein secretion capability with ecological and pathogenic implications (Cianciotto and White, 2017). Collectively, this variability suggests that even within a single production environment, *E. coli* strains can differ substantially in pathogenic potential, with some carrying clear genomic signatures of clinically relevant pathotypes and others appearing more environmentally adapted.

Antimicrobial resistance remains a critical challenge in both human and veterinary medicine (Boerlin et al., 2005). The only core ARG detected across isolates was the multidrug efflux pump gene *mdf(A)*, present in 18/19 strains; this gene confers resistance to multiple drug classes, including aminoglycosides and macrolides, and its ubiquity highlights efflux mechanisms as a foundational resistance strategy in Gram-negative bacteria (Edgar and Bibi, 1997; Swick et al., 2011). This genomic pattern was mirrored phenotypically, with 32% of isolates resistant to streptomycin and 74% resistant to erythromycin, indicating reasonable genotype–phenotype concordance for baseline resistance. The β-lactamase gene *bla*_TEM − 1B_ was the second-most prevalent ARG (58% of strains) and was closely associated with high ampicillin resistance (95%), underscoring ampicillin resistance as a dominant feature of this isolate collection. Notably, 42% of strains carried genes spanning multiple antibiotic classes simultaneously -*bla*_TEM − 1B_ (β-lactams), *qnrS1* (quinolones), *tet(A)* (tetracyclines), and aminoglycoside-modifying enzymes (*aph3*′*)-Ia_3, aac(3)-IId_1*)—consistent with the 26% of isolates exhibiting phenotypic multidrug resistance (to ampicillin, streptomycin, erythromycin, and, in one isolate, gentamicin) across sheep-, goat-, and manure-derived strains from the North-West Province. This pattern adds to growing evidence that agricultural antibiotic use drives the accumulation of MDR determinants in livestock-associated *E. coli* (Laxminarayan et al., 2013; Van Boeckel et al., 2017), and together these results point to the need for integrated surveillance that tracks both genotypic and phenotypic resistance to inform evidence-based antibiotic stewardship policy in livestock systems.

Plasmids play a central role in this resistance landscape by facilitating the acquisition and onward transfer of clinically relevant traits in response to environmental and anthropogenic pressures, including intensive antibiotic use in agriculture and medicine (Götz et al., 1996; Shintani et al., 2015). A total of 71 plasmids were identified across the 19 genomes, comprising 17 conjugative, seven mobilizable, and seven non-mobilizable ARG-carrying plasmids. Conjugation offers bacteria a highly efficient route for disseminating resistance and virulence determinants both within and across species (Guglielmini et al., 2011), but even non-conjugative plasmids can spread ARGs indirectly through mobilization by co-resident mobile elements, demonstrating multiple mechanisms by which resistance genes are disseminated. Plasmid GC content offered further insight into these dynamics: eighteen plasmids deviated from their host genome's GC content by more than 5%, a feature associated with relatively recent acquisition, whereas plasmids with GC content closer to the host chromosome likely reflect longer co-evolutionary histories (Lawrence and Ochman, 1998; Rocha and Danchin, 2002). Consistent with broader trends, most plasmids had slightly lower GC content than their host chromosomes, potentially reflecting the higher energetic cost of G and C nucleotides (Nishida, 2012; Shintani et al., 2015).

The ARG repertoire on plasmids closely mirrored that of the genomes overall, with *bla*_TEM − 1B_ and *tet (A)* each detected on plasmids in 58% of strains, correlating with the high phenotypic resistance to ampicillin (95%) and the moderate-to-high resistance to streptomycin/erythromycin (32–74%) noted above, and reinforcing plasmids as key reservoirs and vehicles for AMR in this dataset. The extended-spectrum β-lactamase (ESBL) gene *bla*_CTX − M−14_ was identified on both the chromosome and a plasmid of strain Ec_GXF23, flagging this isolate as a potentially significant contributor to ESBL dissemination, consistent with the well-established role of plasmids in driving ESBL spread among Enterobacteriaceae (Paterson, 2006). This plasmid also carried *fosA3_1*, the plasmid-mediated fosfomycin-resistance gene, so that a single mobile element in this strain carried both an ESBL determinant and a transferable fosfomycin-resistance determinant. Internationally, co-carriage of *bla*_CTX − M_ variants with *fosA3* on transmissible, largely IncF-family plasmids has been repeatedly documented among *E. coli* from companion animals, poultry, and other food-producing animals in Asia and South America, and in a chicken-derived isolate carrying *bla*_CTX − M−14_ itself alongside *fosA3* on a single plasmid in China (Hou et al., 2012; Yang et al., 2014; Cyoia et al., 2019). In these settings, co-selection by cephalosporins or aminoglycosides is thought to maintain *fosA3* even where fosfomycin itself is not directly used. In South Africa, *fosA3* and *bla*_CTX − M−14_ have each individually been reported in *E. coli* from cattle, irrigation water and fresh produce, and clinical sources (Montso et al., 2019; Alonso et al., 2017; Findlay et al., 2023; Richter et al., 2024), and *bla*_CTX − M−14_ spread across Africa has been linked in part to international poultry trade (Alonso et al., 2017); however, to our knowledge, their co-carriage on a single plasmid in an animal-derived isolate has not previously been described in South Africa. Given the limited veterinary use of fosfomycin in South Africa, persistence of *fosA3* in this strain is more plausibly maintained through co-selection by β-lactams or other antimicrobials than by direct fosfomycin exposure, illustrating how plasmid-linked resistance gene clusters can sustain individual determinants beyond the selective pressure of their originally corresponding drug class, with implications for surveillance of both ESBL and fosfomycin resistance. Two notable plasmid backbones illustrate this further: a mobilizable IncHI1B-type plasmid (AA374) in strain Ec_MG1M3 carried *tet(A)_6, aph(6)-Id_1, aph3*′'*)-Ib_5, sul2_2*, and *bla*_TEM − 1A_1_, conferring resistance to tetracyclines, aminoglycosides, sulfonamides, and β-lactams; and a mobilizable IncR-type plasmid (AB193) in strain Ec_S6_1 carried a similar profile plus *floR_2* (chloramphenicol resistance). IncHI1B plasmids are large, conjugative, and frequently associated with epidemic lineages of *Salmonella enterica* and multidrug-resistant *E. coli*, with stable backbones that support long-term maintenance across diverse hosts and complex multidrug resistance regions embedded in transposons and integrons that enable co-selection under antimicrobial pressure (Johnson and Nolan, 2009; Rozwandowicz et al., 2018). IncR plasmids, although often cryptic and lacking complete self-transmissible machinery, frequently acquire ARGs through recombination and persist via co-resident insertion sequences and transposons, acting as evolutionary “assembly hubs” for resistance gene accumulation and increasingly reported in both clinical and agricultural multidrug-resistant Enterobacteriaceae (Fang et al., 2024; Zhang et al., 2025). In this study, the co-occurrence of aminoglycoside, tetracycline, and β-lactamase genes on both plasmid types reflects strong selective pressure within livestock-associated environments, while the additional presence of *floR* on the IncR plasmid suggests an expanded resistance profile potentially linked to veterinary florfenicol use. Such co-localization of multiple ARG classes raises the risk of co-selection, whereby exposure to a single antimicrobial class can maintain resistance to unrelated drug classes (Carattoli, 2013), a dynamic with direct relevance to manure-mediated and environmental dissemination (Berendonk et al., 2015; Xie et al., 2018).

Plasmid replicon typing revealed diverse incompatibility groups, most prevalently po111_1 (53%), IncF (32%), IncFIC (21%), and IncHI1B (21%). The prominence of IncF-family plasmids is notable given their established association with virulence trait carriage in *E. coli*, particularly within ExPEC and avian pathogenic *E. coli* (APEC) lineages (Johnson and Nolan, 2009; Pitout and Chen, 2023); studies of MDR *E. coli* from Pretoria and from pigs in South Africa similarly report that most resistance determinants and many virulence genes are located on IncF-replicon-containing contigs (Founou et al., 2022). The IncHI plasmids identified here (14%) carried determinants for β-lactams, tetracyclines, aminoglycosides, and fosfomycin, consistent with their recognized role in MDR dissemination, while a smaller fraction (4%) carried IncQ replicons, associated with broad-host-range plasmids capable of stable maintenance across phylogenetically diverse bacteria (Götz et al., 1996; De Gelder et al., 2008). The presence of tRNA copies (1–4 per plasmid) in 14% of plasmids further points to mechanisms supporting long-term plasmid stability across hosts. Taken together, the combination of conjugative and mobilizable plasmids, broad-host-range replicons, and co-localized virulence and resistance determinants identifies plasmids as the primary reservoirs of MDR genes in this livestock-associated *E. coli* population, with corresponding implications for plasmid-mediated AMR spillover into human and environmental bacterial communities within a One Health framework.

Beyond plasmids, other MGE classes contributed further layers of genomic mobility. Transposon-associated genes were prominently represented, with *tnpA, tnpB*, and *tnpC* detected across multiple isolates; these transposases catalyse DNA excision and reintegration, contributing to genome rearrangement and the mobilization of accessory genes (Shkumatov et al., 2022). The frequent occurrence of *tnpB* (74% of isolates) is consistent with large-scale genomic surveys reporting that over 80% of *E. coli* genomes harbor *tnpB*-containing mobile elements, including those of the IS605 family (Shkumatov et al., 2022), and indicates extensive transposon-mediated genome plasticity (Marquart et al., 2024). The co-detection of *rayT, insG*, and *istA* further suggests active regulation of insertion sequences and fine-scale genome modification, reinforcing transposition as a central driver of genomic fluidity in these agricultural isolates.

Prophages, which are known to encode virulence factors, stress-response elements, and regulatory genes that enhance bacterial fitness in both host and environmental contexts (Rousset et al., 2021), were also commonly detected, with *ybfD* and *ybfQ* present in 42% and 32% of isolates, respectively, contributing to genome plasticity, horizontal gene transfer, and the acquisition of adaptive traits such as antibiotic resistance and stress response (Liao et al., 2024). The co-occurrence of prophage elements such as *intS* alongside phage-defense genes (*zorA2–zorE*) suggests a dynamic balance between prophage acquisition and phage-resistance mechanisms, reflecting ongoing co-evolution between *E. coli* and bacteriophages that may be particularly pronounced in manure-rich environments where phage abundance is high.

Integrative and conjugative elements (ICEs) represented the second most prevalent MGE category, with conjugative transfer genes such as *trbL, traA–traQ*, and *mobI* widely distributed across isolates. These genes encode the structural and functional machinery required for pilus formation, mating-pair stabilization, and DNA transfer (Zatyka and Thomas, 1998), and the *tra* operon together with *trbL* is highly conserved across F-like and other conjugative plasmids in *E. coli*, enabling efficient DNA transfer (Koraimann, 2018). The widespread occurrence of *traI* (74% of isolates) points to extensive potential for accessory-trait dissemination via conjugation, underscoring the species' broader capacity for horizontal gene transfer and resistance spread. The mobilization gene *mobI*, detected in six isolates, is typically associated with mobilizable plasmids that depend on co-resident conjugative plasmids for transfer (Ares-Arroyo et al., 2023).

This study offers valuable genomic insights into *E. coli* found in small ruminants and manure environments in South Africa; however, several limitations should be acknowledged. The number of viable isolates retrieved from the biobank was relatively low, which may have constrained the representation of local genomic diversity and limited inter-site comparisons. Not all presumptive isolates underwent whole-genome sequencing, reducing the depth of genomic analysis across the broader collection. While the genomic data provided strong predictions of plasmid-associated and mobilizable elements, no assays were conducted to validate the functional transferability of these plasmids, and reliance on short-read sequencing restricted our ability to reconstruct complete plasmid architectures or resolve complex genomic rearrangements, which would require long-read sequencing approaches. Future studies employing larger, more representative sampling, long-read sequencing, and functional validation are needed to further clarify the dynamics of mobile genetic elements and the dissemination of antimicrobial resistance at the livestock–environment interface.

## 5 Conclusion

This study has provided a comprehensive genomic characterization of *E. coli* isolated from sheep, goats, and manure in South Africa, revealing substantial genomic diversity, extensive mobilome complexity, and widespread antimicrobial resistance. The co-occurrence of globally disseminated sequence types (ST10, ST155) with novel or rarely reported STs highlights both clonal expansion and microevolution within small ruminant populations. The strong association between virulence determinants, resistance genes (e.g., *bla*_TEM − 1B_, *tet (A), mdf (A)*), and diverse mobile genetic elements, including conjugative plasmids (IncF, IncHI1B, IncR), integrative and conjugative elements, and prophages, demonstrates that horizontal gene transfer is a major driver of adaptation and pathogenic potential in these populations. This plasmid and transposon-mediated genomic fluidity positions small ruminant environments as important reservoirs for multidrug-resistant and virulent *E. coli* strains, with the potential to disseminate across human, animal, and environmental interfaces. These findings advance current understanding of *E. coli* evolution in African livestock systems and highlight the need for genomic surveillance within the “One Health” framework to track the emergence of resistance and virulence determinants. The study substantially expands current knowledge of the genomic diversity and mobilome of livestock-associated *E. coli* in South African small-ruminant production systems.

## Data Availability

The genome sequences for the isolates are available in the National Center for Biotechnology and Information (NCBI) database under the BioProject PRJNA1264676, listed under the title “Genome sequencing of E. coli from ruminants in North West Province.
