# Supplementary material for: Genomic insight into the antibiotic-resistant determinants of Escherichia coli from small ruminants and manure in North West Province, South Africa
Source: Front Microbiol. 2026 Sep 28;17:1950193. doi: 10.3389/fmicb.2026.1950193 (PMC13655071; doi:10.3389/fmicb.2026.1950193)
Supplement: Supplementary file 1 [file Supplementary_file_1.docx]

**Genomic insight into the antibiotic-resistant determinants of *Escherichia coli* from small ruminants and manure in North West Province, South Africa**

**Jody Howard^1^, Oriel Thekisoe ^1^, Tsepo Ramatla^2^, Kgaugelo E. Lekota^1*^**

^1^ Unit for Environmental Sciences and Management, North-West University, Potchefstroom, 2531, South Africa.

^2^ Centre for Applied Food Safety and Biotechnology, Department of Life Sciences, Central University of Technology, 1 Park Road, Bloemfontein, 9300, South Africa.

**Corresponding author*

Kgaugelo Lekota, PhD

Tell: +27 18 299 2381, Email: [Lekota.Lekota@nwu.ac.za](mailto:Lekota.Lekota@nwu.ac.za)

Supplementary Table S1: Antibiotic resistance profiles of 19 *E. coli* isolates obtained from faecal sheep and goats as well as manure samples collected in Ventersdorp and Matlwang.

|  |  | ***Escherichia coli* strains (n = 19)** | | |
| --- | --- | --- | --- | --- |
| **Antibiotic** | Class | R (%) | I (%) | S (%) |
| **Ampicillin** | β-lactamase | 18(95) | 0 | 1 (5) |
| **Streptomycin** | Aminoglycoside | 6(32) | 4(21) | 9(47) |
| **Erythromycin** | Macrolide | 14(74) | 4(21) | 1(5) |
| **Gentamycin** | Aminoglycoside | 1(5) | 0 | 18(95) |
| **Ceftriaxone** | Cephalosporin | 0 | 0 | 19(100) |
| **Meropenem** | Carbapenem | 0 | 0 | 19(100) |

**Supplementary Table S2**: Overview of plasmid features identified in 19 *E. coli* strains isolated from small ruminants and manure.

|  | **Plasmids** | **Size (bp)** | **GC content**  **(%)** | **Number of contigs** | **CDS** | **tRNA** | **Replicon types** | **ARGs present** | **Predicted mobility** | **Circularity status** |
| --- | --- | --- | --- | --- | --- | --- | --- | --- | --- | --- |
| **Ec_R8-1** | AA176 | 1 501 20 | 51.09 | 17 | 159 | - | *IncFIA*  *IncFIB*  *IncQ1* | *sul2_14, bla*_TEM-1B_1_*, aph(3')-Ia_1, aph(6)-Id_1, aph(3'')-Ib_5* | Conjugative | Incomplete |
|  | AA474 | 99 711 | 50.89 | 7 | 107 | - | *IncI-gamma/K1* | *dfrA17_1, aadA5_1*  *tet(A)_6* | Conjugative |  |
|  | AA974 | 5 231 | 46.36 | 1 | 4 | - | *Col156* | *-* | Mobilizable |  |
|  | AE974 | 11 876 | 42.03 | 1 | 11 | - | **-** | **-** | Non-mobilizable |  |
| **Ec_GRF5** | AA147 | 5 451 | 50.98 | 1 | 7 | - | *rep_cluster_2401* | *aph(3')-Ia_9* | Mobilizable | Incomplete |
|  | AA303 | 1 383 46 | 50.48 | 16 | 169 | 1 | *IncFIA*  *IncFIC*  *IncFIB*  *IncQ1* | *sul1_5, dfrA7_5*  *catA1_1, bla*_TEM-1B_1_  *aph(6)-Id_1, aph(3'')-Ib_5*  *sul2_3* | Conjugative |  |
| **Ec_GXF23** | AA315 | 51 298 | 51.4 | 2 | 56 | 1 | *rep_cluster_2244* | *-* | Conjugative | Incomplete |
|  | AA374 | 78 564 | 48.01 | 9 | 79 | *-* | *IncHI1B* | *fosA3_1, bla*_CTX-M-14_1_  *bla*_TEM-1B_1_ | Non-mobilizable | Circular |
| **Ec_GXF26** | AA176 | 86 082 | 48.49 | *9* | 75 | - | *IncFIB* | *-* | Mobilizable | Incomplete |
|  | AA474 | 1 287 64 | 50.53 | 5 | 145 | 1 | *IncFIC*  *rep_cluster_2244*  *IncI-gamma/K1* | *tet(A)_6, bla*_TEM-1B_1_ | Conjugative |  |
|  | AD484 | 4 617 | 44.1 | 1 | 4 | - | *rep_cluster_2350* | *-* | Mobilizable |  |
| **Ec_M2K2** | AA304 | 1 072 39 | 49.89 | 7 | 113 | 2 | *IncFIA*  *IncFIB* | *bla*_TEM-1A_1_ | Conjugative | Incomplete |
|  | AB192 | 29 523 | 55.66 | 4 | 30 | - | *IncR* | *floR_2* | Non-mobilizable |  |
| **Ec_MG1M3** | AA374 | 87 580 | 48.74 | 35 | 86 | - | *IncHI1B* | *tet(A)_6*  *aph(6)-Id_1, aph(3'')-Ib_5, sul2_2*  *bla*_TEM-1A_1_ | Mobilizable | Incomplete |
|  | AB193 | 18 927 | 52.44 | 4 | 19 | - | *IncR* | *-* | Non-mobilizable | Incomplete |
|  | AC621 | 47 982 | 44.49 | 7 | 69 | 1 | **-** | **-** | Non-mobilizable | Incomplete |
|  | AC527 | 2 862 | 48.04 | 1 | 4 | 1 | *rep_cluster_2373* | *-* | Non-mobilizable | Incomplete |
|  | Unclassified plasmid | 1 764 | 50.06 | 1 | 1 | - | *Col(MG828)* | *-* | Mobilizable | Circular |
| **Ec_R2-1** | AA551 | 31 510 | 50 | 3 | 40 | - | *IncN* | *-* | Conjugative | **-** |
|  | AC305 | 31 943 | 43 | 2 | 43 | - | *IncX1* | *aph(3')-Ia_3* | Conjugative |  |
| **Ec_S1_1A** | AA374 | 1 173 08 | 47.75 | 19 | 123 | - | *IncHI1B* | *bla*_TEM-1B_1_ | Mobilizable | Incomplete |
|  | AB687 | 1 505 | 50.83 | 1 | 1 | - | *Col(MG828)* | *-* | Mobilizable | Circular |
|  | AC770 | 5 985 | 45.58 | 1 | 10 | - | **-** | **-** | Non-mobilizable | Incomplete |
|  | AD672 | 19 228 | 53.54 | 7 | 24 | - | **-** | **-** | Non-mobilizable | Incomplete |
|  | Unclassified plasmid | 1 764 | 49.15 | 1 | 1 | - | *Col(MG828)* | *-* | Mobilizable | Circular |
| **Ec_S1_1B** | AA374 | 1 171 67 | 47.72 | 17 | 118 | - | *IncHI1B* | *bla*_TEM-1B_1_ | Mobilizable | Incomplete |
|  | AD672 | 2 236 5 | 54.17 | 5 | 27 | - | **-** | **-** | Non-mobilizable | Incomplete |
|  | Unclassified plasmid | 1 764 | 50.74 | 1 | 1 | - | *Col(MG828)* | *-* | Mobilizable | Circular |
| **Ec_S5-1** | AA302 | 96 109 | 49.99 | 1 | 92 | 2 | *IncFIA*  *IncFIB*  *IncFIC* | *bla*_TEM-1B_1_  *tet(A)_6* | Conjugative | Circular |
|  | AD672 | 16 025 | 54.52 | 6 | 19 | - | **-** | **-** | Non-mobilizable | Incomplete |
| **Ec_S6_1** | AB193 | 66 174 | 54.67 | 11 | 79 | - | *IncR* | *floR_2,*  *tet(A)_6*  *aph(6)-Id_1, aph(3'')-Ib_5*  *sul2_2, bla*_TEM-1A_1_ | Mobilizable | Incomplete |
| **Ec_S16** | AA474 | 98 538 | 50.27 | 9 | 109 | - | *IncI-gamma/K1* | *-* | Conjugative | Incomplete |
|  | AA977 | 5 290 | 47.2 | 1 | 5 | - | *Col156* | *-* | Mobilizable |  |
|  | AD446 | 7 976 | 51.25 | 3 | 10 | - | **-** | **-** | Non-mobilizable |  |
|  | AF190 | 9 063 | 54.85 | 2 | 10 | - | **-** | **-** | Non-mobilizable |  |
|  | AG294 | 19 833 | 52.6 | 5 | 15 | - | **-** | **-** | Non-mobilizable |  |
|  | AH321 | 2 326 | 51.12 | 1 | 4 | - | **-** | **-** | Non-mobilizable |  |
| **Ec_S18_1A** | AA175 | 1 100 66 | 50.85 | 6 | 106 | - | *IncFIA* | *bla*_TEM-1B_1_  *aac(3)-IId_1* | Conjugative | Incomplete |
|  | AA179 | 18 858 | 43.19 | 2 | 19 | - | *IncFIB* | *-* | Non-mobilizable |  |
|  | AA372 | 58 410 | 42.41 | 3 | 76 | - | *IncI2* | *-* | conjugative |  |
|  | AA374 | 79 877 | 46.61 | 11 | 71 | - | *IncHI1B* | *-* | Non-mobilizable |  |
|  | AD158 | 33 251 | 47.94 | 3 | 48 | - | *rep_cluster_1760* | *-* | Conjugative |  |
|  | AF232 | 3 347 | 54.83 | 2 | 3 | - | **-** | **-** | Non-mobilizable |  |
| **Ec_S20-1** | AA374 | 1 177 18 | 47.76 | 20 | 123 | - | *IncHI1B* | *bla*_TEM-1B_1_ | mobilizable | Incomplete |
|  | AC770 | 5 985 | 45.58 | 1 | 10 | - | **-** | **-** | Non-mobilizable | Incomplete |
|  | Unclassified plasmid | 1 764 | 50.74 | 1 | 1 | - | *Col(MG828)* | *-* | Mobilizable | Circular |
| **Ec_S38** | AA314 | 59 529 | 52.06 | 4 | 63 | 1 | *IncFIA*  *IncFIC* | *-* | conjugative | Incomplete |
|  | AA374 | 1 048 17 | 47.39 | 18 | 109 | - | *IncHI1B* | *-* | Mobilizable | Incomplete |
|  | AA974 | 4 726 | 47.25 | 1 | 5 | - | *Col156* | *-* | Mobilizable | circular |
|  | AB947 | 5 932 | 55.04 | 1 | 8 | - | **-** | *bla*_TEM-1B_1_ | Non-mobilizable | Incomplete |
|  | AD690 | 57 474 | 45.55 | 4 | 50 | - | **-** | **-** | Non-mobilizable | Incomplete |
|  | AF232 | 6 266 | 52.92 | 1 | 4 | - | **-** | **-** | Non-mobilizable | Incomplete |
|  | Unclassified plasmid | 5 933 | 46.2 | 1 | 4 | - | *rep_cluster_2350* | *-* | Mobilizable | circular |
|  | Unclassified plasmid | 3 520 | 47.7 | 1 | 3 | - | *rep_cluster_2335* | *-* | Non-mobilizable | circular |
| **Ec_S18_1B** | Unclassified plasmid | 10 752 | 47.43 | 1 | 9 | - | *IncHI1B* | *-* | Non-mobilizable | Incomplete |
| **Ec_GXF14** | AA374 | 87 343 | 47.12 | 4 | 94 | *-* | *IncHI1B* | *bla*_TEM-106_1_ | Non-mobilizable | Incomplete |
| **Ec_GXF8** | AA372 | 60 476 | 42.5 | 5 | 84 | *-* | *IncI2* | *-* | conjugative | Incomplete |
|  | AA378 | 60 476 | 47.5 | 11 | 97 | 3 | *IncHI1B* | *-* | Non-mobilizable |  |
|  | AB002 | 86 617 | 47.36 | 7 | 102 | 4 | *rep_cluster_1704* | *-* | Non-mobilizable |  |
|  | AC082 | 42 181 | 54.55 | 16 | 46 | *-* | *-* | *dfrA7_5*  *bla*_TEM-1B_1_  *sul1_5* | Non-mobilizable |  |
|  | AC120 | 7 643 | 49.17 | 3 | 6 | *-* | *IncQ1* | *sul2_3* | Non-mobilizable |  |
|  | AC662 | 74 736 | 49.12 | 3 | 86 | **-** | **-** | **-** | conjugative |  |
|  | AD446 | 1 600 5 | 52.51 | 3 | 13 | **-** | **-** | *-* | Non-mobilizable |  |
|  | AD672 | 10 375 | 55.73 | 3 | 10 | **-** | **-** | **-** | Non-mobilizable |  |
|  | AF232 | 6 344 | 49.73 | 1 | 6 | **-** | **-** | **-** | Non-mobilizable |  |
|  | AG271 | 1 412 | 54.67 | 1 | 2 | **-** | **-** | **-** | Non-mobilizable |  |
|  | AG294 | 30 577 | 52.17 | 5 | 24 | **-** | **-** | **-** | Non-mobilizable |  |
|  | AH321 | 1470 | 55.44 | 1 | 1 | **-** | **-** | **-** | Non-mobilizable |  |
| **Ec_GXF1** | AA175 | 99192 | 48.97 | 20 | 104 | - | *IncFIB*  *IncFIC*  *rep_cluster_2244* | *bla*_TEM-106_1_  *tet(A)_6* | Mobilizable | Incomplete |
|  | AA329 | 74071 | 52.86 | 12 | 85 | *-* | *IncFIA*  *IncFIC* | *-* | Conjugative |  |
|  | AC313 | 16200 | 52.93 | 5 | 17 | **-** | **-** | *qnrS1_1* | Non-mobilizable |  |

**Supplementary Figure S1**: Antibiotic resistance profiles of 19 *E. coli* isolates identified in faeces (sheep and goats) and manure from Ventersdorp and Matlwang using Kirby-Bauer Disk Diffusion.

**Key:**

_SV = Sheep Ventersdorp

_SM = Sheep Matlwang

_GV = Goats Ventersdorp

_MV = Manure Ventersdorp

**Supplementary Figure S2:** Circular plot of coding sequences found in plasmid AA303 identified in strain Ec_GRF5 of phylogroup D.

**Supplementary Figure S3:** Circular plot of a multidrug-resistant plasmid AA374 identified in strain Ec_MG1M3.

**Supplementary Figure S4**: Circular plot of a multidrug resistant plasmid AB193 identified in strain Ec_S6_1.

**Supplementary Figure S5**: Plasmid replicon types determined among the 19 *E. coli* strains using ResFinder. Heatmap: Dark blue – Present; Light blue – Absent.
